# Supplementary material for: A phase 1 randomized, placebo-controlled trial of a combination typhoid and non-typhoidal Salmonella polysaccharide conjugate vaccine
Source: medRxiv. 2025 Sep 18:2025.09.15.25335795. Preprint. [Version 1] doi: 10.1101/2025.09.15.25335795 (PMC12458498; doi:10.1101/2025.09.15.25335795)
Supplement: Supplement 1 [file media-1.pdf]

Supplementary Appendix

Article Title: A phase 1 randomized, placebo-controlled trial of a combination typhoid and non-typhoidal *Salmonella* polysaccharide conjugate vaccine

Authors: Wilbur H. Chen, Robin Barnes, Michael J. Sikorski, Reva Datar, Roohali Sukhavasi, Yuanyuan Liang, Rekha R. Rapaka, Marcela F. Pasetti, Marcelo B. Sztein, Rezwanul Wahid, Sharon M. Tennant, Raphael Simon, Scott M. Baliban, James E. Galen, Andrew Lees, Biana Bernshtein, Galit Alter, Raches Ella, Krishna Mohan, M. Gangadhara Naidu, D. Yogewar Rao, Krishna M. Ella, Myron M. Levine

Table of Contents

Figure 1: CONSORT Diagram 2

Table 1. Baseline Demographic Characteristics 3

Table 2. Solicited Adverse Events 4

Figure 2. Local Solicited Adverse Events 5

Figure 3. Systemic Solicited Adverse Events 6

Figure 4. Clinical Safety Laboratories 7

Table 3: Serum ELISA IgG and IgA responses 8

Figure 5. Persistence of serum antigen-specific IgG responses 9

Figure 6. Persistence of serum antigen-specific IgA responses 10

Table 4. Unsorted Antibody Secreting Cells Responses 11

Table 5: Sorted Antibody Secreting Cells Responses, for Tissue Homing Potential 12

Table 6. Memory B Cell Responses 13

Figure 7. Gating Protocol of Sorted B cell subpopulations 15

Figure 8. Percentage of antigen-specific BM responses 16

Figure 1: CONSORT Diagram

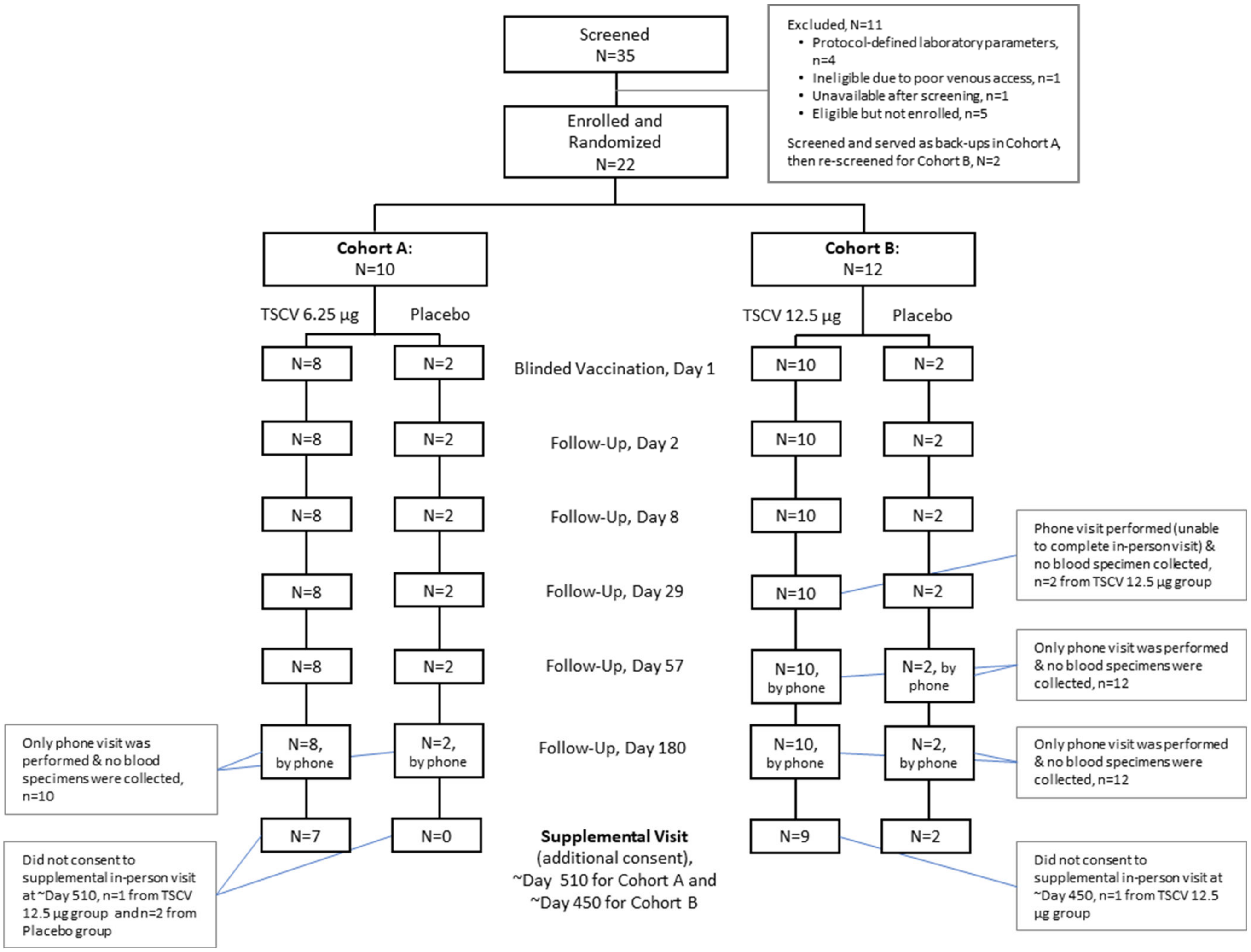

Table 1. Baseline Demographic Characteristics

By Cohort

	Cohort A TSCV 6.25 μg or placebo	Cohort B TSCV 12.5 μg or placebo	All Subjects
Total Enrolled	10	12	22
Gender,			
No. male (%)	2 (20%)	3 (25%)	5 (22.7%)
No. female (%)	8 (80%)	9 (75%)	17 (77.3%)
Age, year			
Mean (SD)	31.6 (8.44)	31.6 (9.39)	31.6 (8.75)
Median [Q1, Q3]	30.5 [23.5, 39.25]	31 [25.25, 38.5]	31 (23.5, 39.5)
Min, Max	22, 44	18, 45	18, 45
Ethnicity,			
No. non-Hispanic (%)	10 (100%)	12 (100%)	22 (100%)
Race, n (%)			
Black/African American	6 (60%)	6 (50%)	12 (54.5%)
White	2 (20%)	5 (42%)	7 (31.8%)
Asian	1 (10%)	0	1 (4.5%)
Am Indian/Alaska Native	0	0	0
Pacific Islander/Hawaiian	0	0	0
Multi-race	1 (10%)	0	1 (4.5%)
Unknown or not reported	0	1 (8%)	1 (4.5%)

By Study Product

	TSCV 6.25 μg	TSCV 12.5 μg	Placebo
Total Enrolled	8	10	4
Gender,			
No. male (%)	1	1 (10%)	3 (75%)
No. female (%)	7	9 (90%)	1 (25%)
Age, year			
Mean (SD)	33.6 (8.21)	32.7 (9.97)	24.7 (1.89)
Median [Q1, Q3]	35 (26.75, 40.25)	35 (24.75, 39.5)	25.5 (24.25, 26)
Min, Max	23, 44	18, 45	22, 26
Ethnicity,			
No. non-Hispanic (%)	8 (100%)	10 (100%)	4 (100%)
Race, n (%)			
Black/African American	5 (71%)	5 (50%)	2 (50%)
White	2 (29%)	4 (40%)	1 (25%)
Asian	0	0	1 (25%)
Am Indian/Alaska Native	0	0	0
Pacific Islander/Hawaiian	0	0	0
Multi-race	1 (14%)	0	0
Unknown or not reported	0	1 (10%)	0

Table 2. Solicited Adverse Events

Maximum Grade Severity over 7-days Post-Vaccination, by Solicited Symptom

	TSCV 6.25 µg				TSCV 12.5 µg				placebo			
**Grade**	**None 0**	**Mild 1**	**Mod 2**	**Sev 3**	**None 0**	**Mild 1**	**Mod 2**	**Sev 3**	**None 0**	**Mild 1**	**Mod 2**	**Sev 3**
Systemic Symptoms												
Fever	8				10				4			
Chills	8				0		1		4			
Fatigue	8				5	3	2		2	2		
Malaise	6	2			8	1	1		4			
Myalgia	6	2			6	1	3		4			
Arthralgia	8				8	2			4			
Nausea	8				8	2			4			
Headache	5	3			7	2	1		4			
Local Symptoms												
Pain	2	6			1	7	2		4			
Erythema	5	3			5	4	1		3	1		
Induration	8				7	2	1		3	1		
Ecchymosis	5	3			9	1			4			

Figure 2. Local Solicited Adverse Events

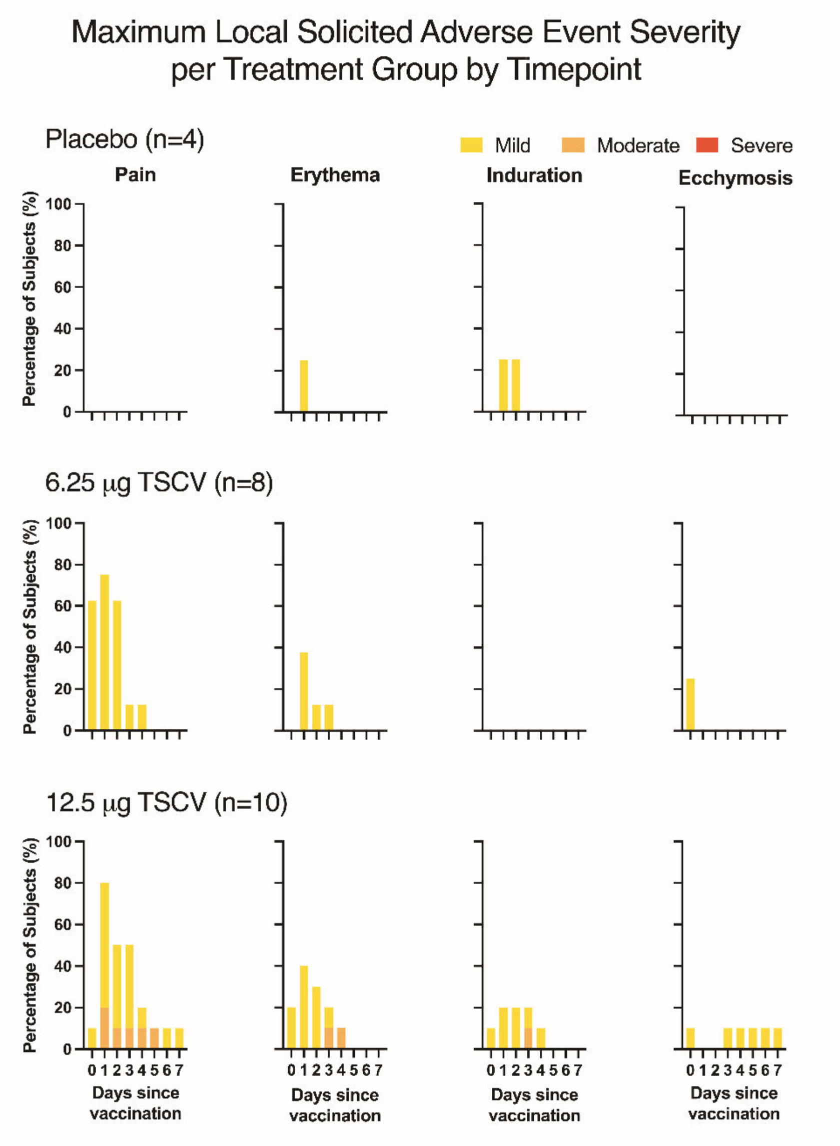

Figure 3. Systemic Solicited Adverse Events

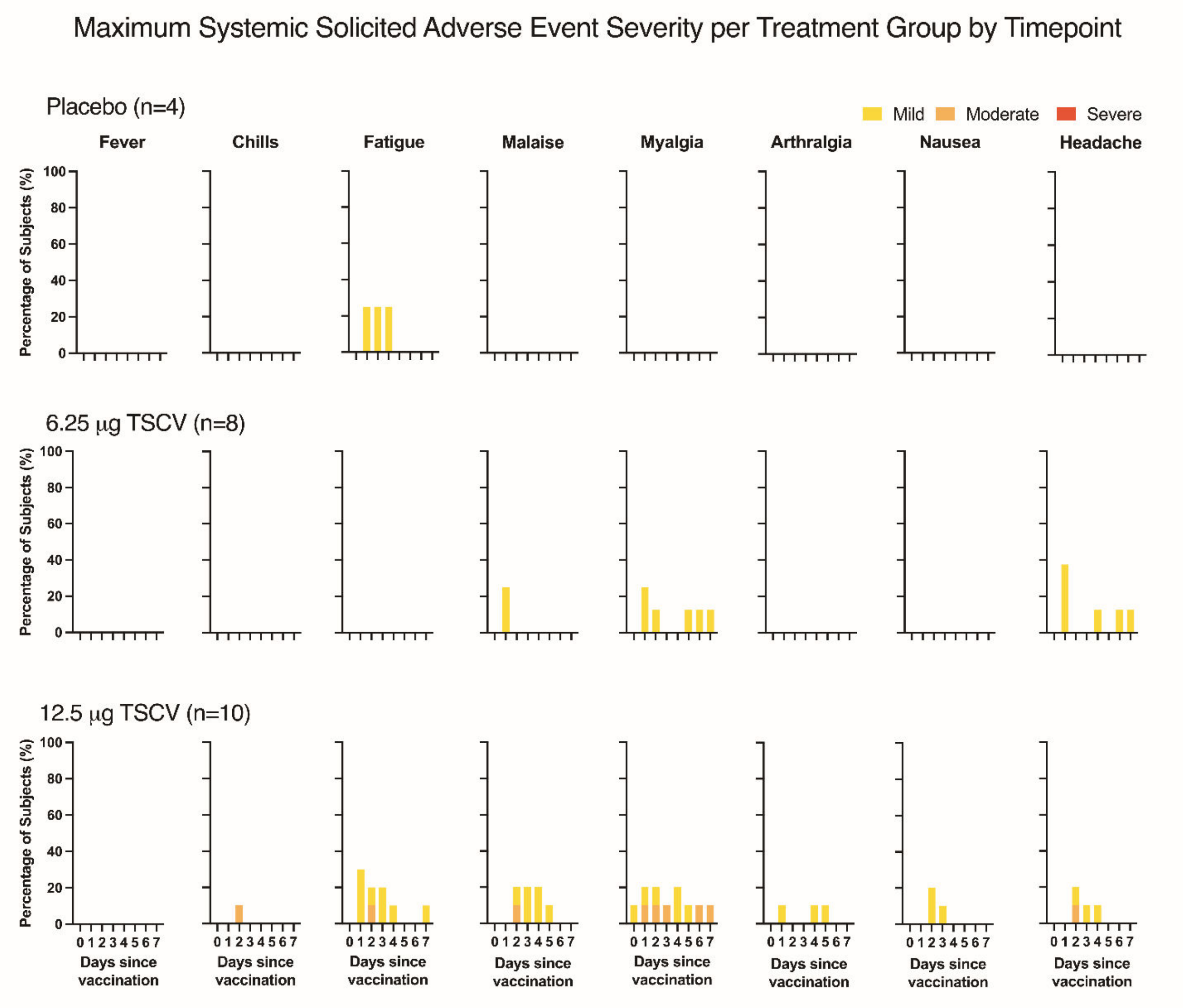

Figure 4. Clinical Safety Laboratories

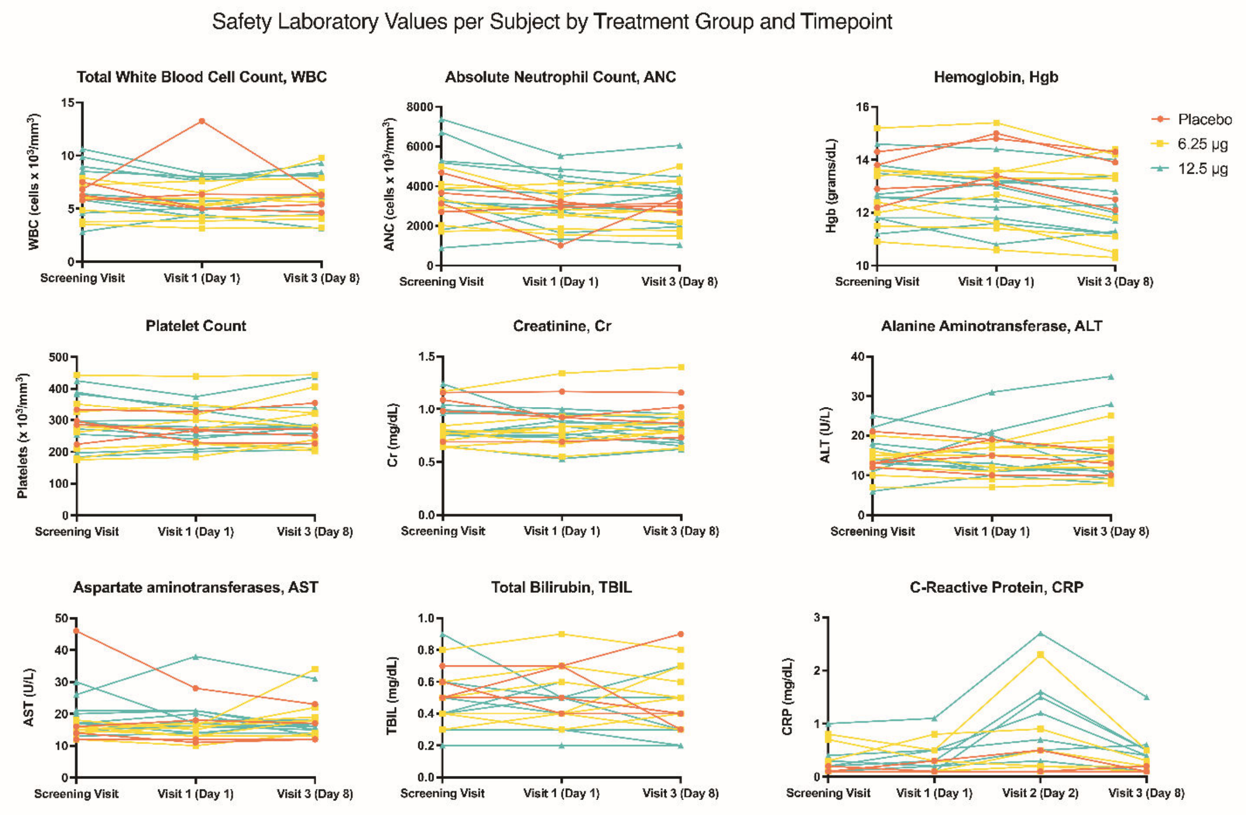

Table 3: Serum ELISA IgG and IgA responses

	S. Enteritidis COPS IgG (EU/mL)						*S.* Enteritidis COPS IgA (EU/mL)					
	placebo		6.25 µg		12.5 µg		placebo		6.25 µg		12.5 µg	
	conv	GMT	conv	GMT	conv	GMT	conv	GMT	conv	GMT	conv	GMT
Day 1	-	137	-	191	-	252	-	41	-	25	-	36
Day 29	0/4	181	8/8	12,190	8/8	21,958	0/4	40	8/8	2,709	8/8	3,501
Day 57	0/2	176	8/8	7,011	ns	ns	0/2	32	8/8	1,239	ns	ns
Day 450/510	0/2	102	5/7	786	5/7	2,596	0/2	45	6/7	450	6/7	594

	S. Typhimurium COPS IgG (EU/mL)						*S.* Typhimurium COPS IgA (EU/mL)					
	placebo		6.25 µg		12.5 µg		placebo		6.25 µg		12.5 µg	
	conv	GMT	conv	GMT	conv	GMT	conv	GMT	conv	GMT	conv	GMT
Day 1	-	1.545	-	556	-	553	-	160	-	153	-	160
Day 29	0/4	1,781	8/8	29,976	8/8	33,456	0/4	199	8/8	13,592	8/8	17,874
Day 57	0/2	2,522	8/8	17,665	ns	ns	0/2	357	7/8	6,633	ns	ns
Day 450/510	0/2	553	3/7	2,398	4/7	2,176	0/2	111	6/7	2,647	7/7	3,205

	S. Typhi Vi IgG (EU/mL)						*S.* Typhi Vi IgA (EU/mL)					
	placebo		6.25 µg		12.5 µg		placebo		6.25 µg		12.5 µg	
	conv	GMT	conv	GMT	conv	GMT	conv	GMT	conv	GMT	conv	GMT
Day 1	-	2.4	-	1.4	-	0.9	-	97	-	82	-	128
Day 29	0/4	2.4	8/8	173	8/8	70	0/4	99	8/8	4,573	7/8	4,470
Day 57	0/2	6.1	8/8	148	ns	ns	0/2	100	8/8	3,420	ns	ns
Day 450/510	0/2	1.0	7/7	50	5/7	19	0/2	88	7/7	1,830	6/7	1,257

	S. Enteritidis FliC IgG (EU/mL)						*S.* Enteritidis FliC IgA (EU/mL)					
	placebo		6.25 µg		12.5 µg		placebo		6.25 µg		12.5 µg	
	conv	GMT	conv	GMT	conv	GMT	conv	GMT	conv	GMT	conv	GMT
Day 1	-	2,369	-	1,106	-	1,238	-	155	-	154	-	18
Day 29	0/4	1,970	7/8	10,687	8/8	18,056	0/4	147	2/8	472	6/8	797
Day 57	0/2	3,277	5/8	5,295	ns	ns	0/2	356	1/8	285	ns	ns
Day 450/510	0/2	1,450	0/7	1,295	0/7	1,803	0/2	65	0/8	188	0/7	221

	S. Typhimurium FliC IgG (EU/mL)						*S.* Typhimurium FliC IgA (EU/mL)					
	placebo		6.25 µg		12.5 µg		placebo		6.25 µg		12.5 µg	
	conv	GMT	conv	GMT	conv	GMT	conv	GMT	conv	GMT	conv	GMT
Day 1	-	2,403	-	874	-	778	-	178	-	122	-	164
Day 29	0/4	1,760	7/8	9,442	8/8	19,795	0/4	179	5/8	527	8/8	1,380
Day 57	0/2	5,136	5/8	4,587	ns	ns	0/2	746	1/8	275	ns	ns
Day 450/510	0/2	970	0/7	1,008	1/7	1,746	0/2	42	0/7	199	1/7	325

conv: number with seroconversion by total number of participants at that timepoint. Seroconversion was defined as four-fold or higher titer than baseline (day 1) titer.
GMT: geometric mean titer
ns: no sample
Note: for Cohort A the last visit was ~Day 510 and for Cohort B the last visit was ~Day 450.

Figure 5. Persistence of serum antigen-specific IgG responses

Individual serum antigen-specific IgG titers, comparing day 1 (baseline) and Day 450-510 titer. Shaded shapes indicate post-vaccination timepoints where there was seroconversion (four-fold or higher titer than baseline titer). Diamond shapes indicate subjects missing the Day 450-510 timepoint.

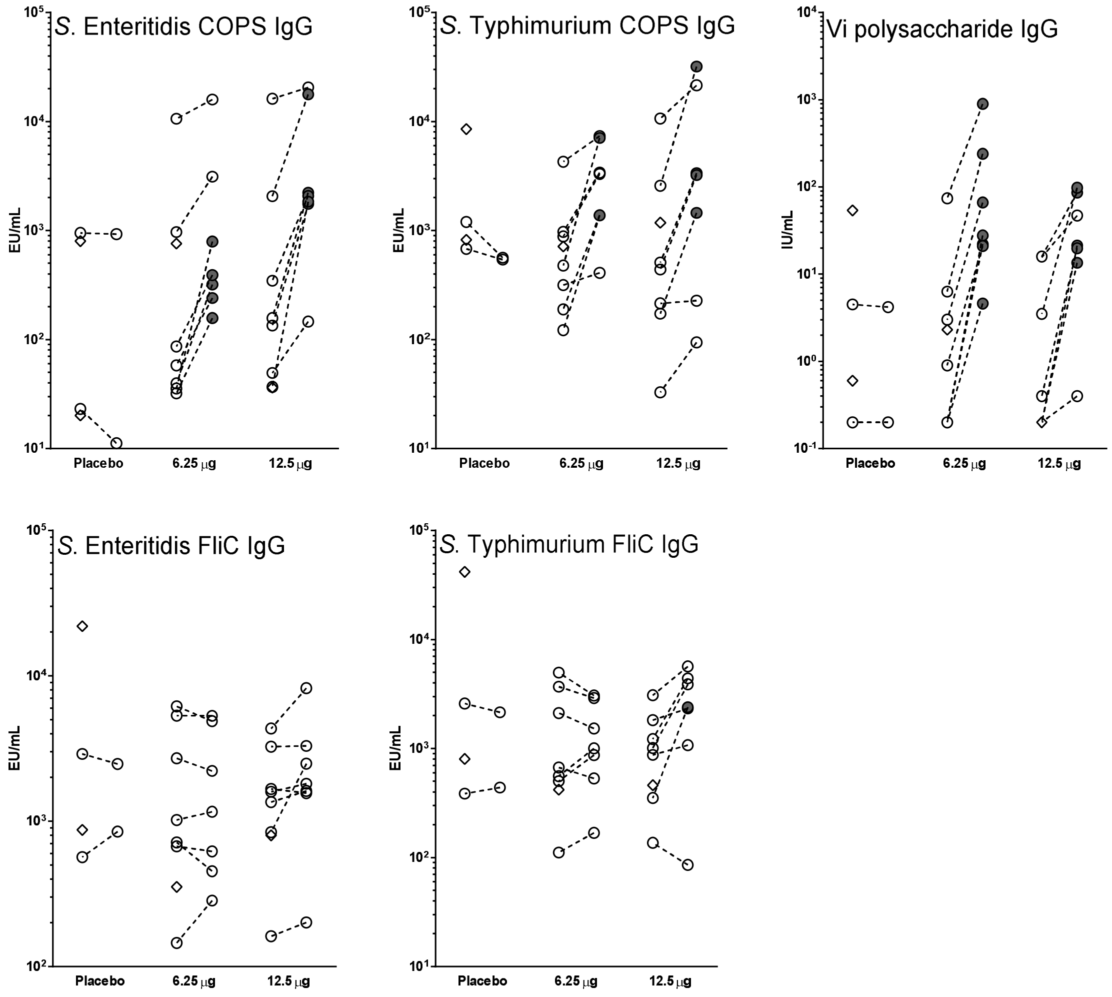

Figure 6. Persistence of serum antigen-specific IgA responses

Individual serum antigen-specific IgA titers, comparing day 1 (baseline) and Day 450-510 titer. Shaded shapes indicate post-vaccination timepoints where there was seroconversion (four-fold or higher titer than baseline titer). Diamond shapes indicate subjects missing the Day 450-510 timepoint.

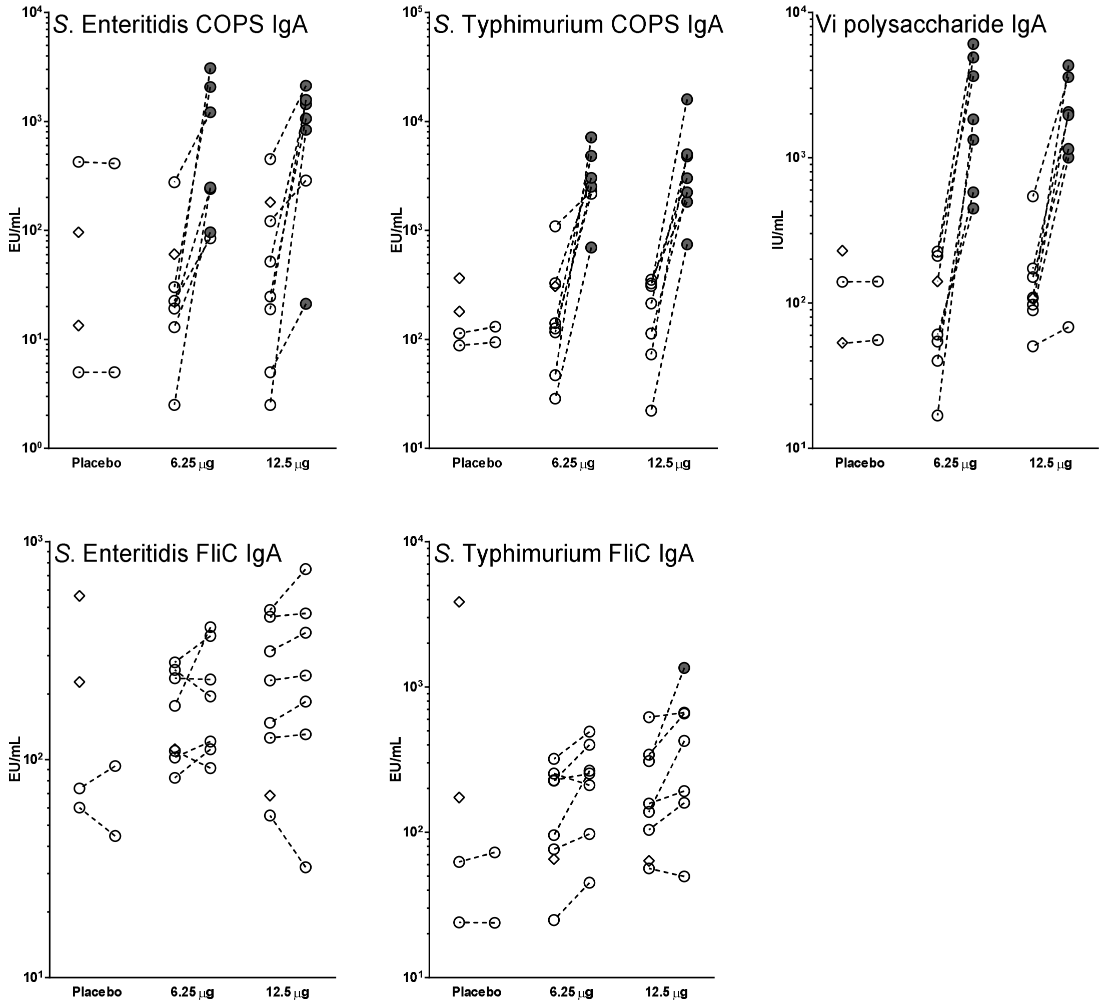

Table 4. Unsorted Antibody Secreting Cells Responses

Unsorted ASC responses were only measured among Cohort B participants.

12.5 µg TSCV, n=10

ASC IgG	SE LPS	STm LPS	Vi	SE FliC	STm FliC	TT
Mean	615	690	163	5	60	310
Median	875	875	80	0	98	428
Range	(0, 875)	(0, 875)	(2.5, 875)	(0, 30)	(0, 188)	(20, 875)
% Responder	90%	90%	80%	20%	80%	100%

ASC IgA	SE LPS	STm LPS	Vi	SE FliC	STm FliC	TT
Mean	524	737	349	8	20	24
Median	491	875	229	4	13	16
Range	(30, 875)	(60, 875)	(28, 875)	(0, 28)	(0, 60)	(0, 65)
% Responder	100%	100%	100%	30%	60%	60%

Placebo, n=2

ASC IgG	SE LPS	STm LPS	Vi	SE FliC	STm FliC	TT
Range	(0, 3)	(0, 0)	(0, 0)	(0, 0)	(0, 0)	(0, 0)
% Responder	0	0	0	0	0	0

ASC IgA	SE LPS	STm LPS	Vi	SE FliC	STm FliC	TT
Range	(0, 0)	(3, 5)	(0, 0)	(0, 0)	(0, 0)	(0, 0)
% Responder	0	0	0	0	0	0

Table 5: Sorted Antibody Secreting Cells Responses, for Tissue Homing Potential

Sorted ASC responses were measured from Cohort B participants. Memory B cells (CD19+ CD27+) were sorted into 4 subpopulations: CD62L+ α4β7- (LN: Lymph Node homing), CD62L- α4β7- (unknown homing), CD62L+ α4β7+ (LN and gut mucosa homing), and CD62L- α4β7+ (gut mucosa homing) from recipients of 12.5µg of TSCV intramuscularly (n=10). Antigen-specific cells detected for each sorted subpopulation were converted to Spot Forming Cells (SFC)/million of purified CD19+CD27+ cells based on the number of sorted cells seeded in each well. Subjects with >500 SFC/million cells in at least 2 out of 4 sorted subsets for each antigen were included in the analysis.

		62L- A4β7- (unknown homing)	62L+ A4β7- (Lymph Node)	62L+ A4β7+ (LN AND Gut Mucosa)	62L- A4β7+ (Gut Mucosa)
**Vi IgG**					
	Mean	2,156	6,723	746	851
	Median	1,135	2,727	833	0
	Range	0-8,250	58-32,432	0-1,429	0-5,641
**Vi IgA**					
	Mean	4,578	6,863	12,670	8,908
	Median	5,000	4,886	13,966	8,622
	Range	0-10,702	1,516-14,148	855-32,222	0-22,167
**SE IgG**					
	Mean	5,610	6,758	8,259	7,738
	Median	4,512	3,472	6,827	5,917
	Range	0-19,000	729-19,773	2,286-19,130	0-24,375
**SE IgA**					
	Mean	6,642	6,278	16,133	22,283
	Median	2,994	5,442	11,467	20,029
	Range	1,000-21,111	2,102-12,923	5,000-37,586	0-45,667
**STm IgG**					
	Mean	5,774	11,349	18,645	13,280
	Median	4,693	12,810	16,251	10,682
	Range	0-18,000	1,166-19,773	3,519-42,391	0-34,667
**STm IgA**					
	Mean	9,380	10,947	31,565	35,115
	Median	8,700	10,923	25,841	37,037
	Range	0-20,556	6,592-17,796	5,600-76,000	0-71,250

Table 6. Memory B Cell Responses

Antigen-specific memory B (B_M) cell responses were measured and are reported by the % of specific B_M per total B cells.

6.25 µg TSCV

B_m IgG Day 1	Vi	SE LPS	STm LPS	SE FliC	STm FliC	TT	SCh LPS
N	8	8	8	7	7	7	7
Mean	0	0.0343	0.009	0.013	0.013	0.160	0
Median	0	0	0	0	0	0.110	0
Range	0, 0	0, 0.190	0, 0.07	0, 0.09	0, 0.9	0, 0.43	0, 0
% Responder	0%	12.5%	0%	0%	0%	71.4%	0%

B_m IgA Day 1	Vi	SE LPS	STm LPS	SE FliC	STm FliC	TT	SCh LPS
N	8	8	8	7	7	7	7
Mean	0	0.106	0.009	0.071	0.131	0.047	0
Median	0	0	0	0	0	0	0
Range	0, 0	0, 0.57	0, 0.07	0, 0.50	0, 0.50	0, 0.33	0, 0
% Responder	0%	25%	0%	14.3%	28.6%	14.3%	0%

B_m IgG Day 29	Vi	SE LPS	STm LPS	SE FliC	STm FliC	TT	SCh LPS
N	8	8	8	7	7	7	6
Mean	0.370	0.128	0.126	0.120	0.130	1.10	0.055
Median	0.285	0.085	0.085	0.070	0.130	0.89	0
Range	0, 1.08	0, 0.34	0, 0.33	0, 0.37	0, 0.32	0.12, 2.47	0, 0.295
% Responder	50%	37.5%	62.5%	14.3%	57.1%	85.7%	16.7%

B_m IgA Day 29	Vi	SE LPS	STm LPS	SE FliC	STm FliC	TT	SCh LPS
N	8	8	8	7	7	7	5
Mean	1.63	0.648	0.126	7	0.123	0	0.042
Median	0.63	0.44	0.085	7	0	0	0
Range	0, 7.15	0, 1.81	0, 0.33	0, 0	0, 0.51	0, 0	0, 0.21
% Responder	75%	75%	37.5%	0%	14.3%	0%	20%

B_m IgG Day 57	Vi	SE LPS	STm LPS	SE FliC	STm FliC	TT	SCh LPS
N	7	8	7	7	7	7	6
Mean	0.210	0.0338	0.093	0.029	0.086	0.81	0
Median	0.230	0	0.07	0	0	0.81	0
Range	0, 0.55	0, 0.17	0, 0.28	0, 0.20	0, 0.32	0.22, 1.39	0, 0
% Responder	57%	12.5%	42.9%	28.6%	28.6%	87.5%	0%

B_m IgA Day 57	Vi	SE LPS	STm LPS	SE FliC	STm FliC	TT	SCh LPS
N	7	7	7	7	7	7	6
Mean	0.71	0.447	0.093	0	0	0	0
Median	0.41	0.56	0.07	0	0	0	0
Range	0, 2.06	0, 0.92	0, 0.28	0, 0	0, 0	0, 0	0, 0
% Responder	71.4%	57.1%	28.6%	0%	0%	0%	0%

B_m IgG D510	Vi	SE LPS	STm LPS	SE FliC	STm FliC	TT	SCh LPS
N	7	7	7	6	6	6	5
Mean	0	0.027	0	0.055	0.020	0.242	0
Median	0	0	0	0	0	0.20	0
Range	0, 0	0, 0.19	0, 0	0, 0.33	0, 0.12	0.12, 0.53	0, 0
% Responder	0%	0%	0%	16.7%	16.7%	50%	0%

B_m IgA D510	Vi	SE LPS	STm LPS	SE FliC	STm FliC	TT	SCh LPS
N	7	6	7	6	6	7	5
Mean	0.104	0.120	0	0	0	0	0
Median	0	0.085	0	0	0	0	0
Range	0, 0.40	0, 0.37	0, 0	0, 0	0, 0	0, 0	0, 0
% Responder	28.6%	33.3%	0%	0%	0%	0%	0%

12.5 µg TSCV

B_m IgG Day 1	Vi	SE LPS	STm LPS	SE FliC	STm FliC	TT	SCh LPS
N	10	10	10	10	10	10	10
Mean	0	0	0	0.02	0.012	0.143	0
Median	0	0	0	0	0	0.105	0
Range	0, 0	0, 0	0, 0	0, 0.16	0, 0.12	0, 0.4	0, 0
% Responder	0%	0%	0%	10%	10%	50%	0%

B_m IgA Day 1	Vi	SE LPS	STm LPS	SE FliC	STm FliC	TT	SCh LPS
N	10	10	10	9	10	10	10
Mean	0.14	0.087	0.100	0	0	0.019	0
Median	0	0	0	0	0	0	0
Range	0, 0.69	0, 0.57	0, 0.56	0, 0	0, 0	0, 0.19	0, 0
% Responder	40%	20%	20%	0%	0%	10%	0%

B_m IgG Day 29	Vi	SE LPS	STm LPS	SE FliC	STm FliC	TT	SCh LPS
N	8	10	8	10	9	8	10
Mean	0.19	0.059	0.10	0.009	0.078	0.55	0
Median	0.145	0	0	0	0	0.3	0
Range	0, 0.5	0, 0.38	0, 0.76	0, 0.09	0.49	0.07, 1.22	0, 0
% Responder	62.5%	30%	12.5%	0%	22.2%	87.5%	0%

B_m IgA Day 29	Vi	SE LPS	STm LPS	SE FliC	STm FliC	TT	SCh LPS
N	8	10	8	10	9	7	10
Mean	1.135	0.344	0.703	0.097	0.064	0	0
Median	0.74	0.21	0.67	0	0	0	0
Range	0, 3.38	0, 107	0.16, 1.38	0, 0.74	0, 0.42	0, 0	0, 0
% Responder	75%	50%	100%	20%	22.2%	0%	0%

B_m IgG Day 57	Vi	SE LPS	STm LPS	SE FliC	STm FliC	TT	SCh LPS
N	ns	ns	ns	ns	ns	ns	ns
Mean							
Median							
Range							
% Responder							

B_m IgA Day 57	Vi	SE LPS	STm LPS	SE FliC	STm FliC	TT	SCh LPS
N	ns	ns	ns	ns	ns	ns	ns
Mean							
Median							
Range							
% Responder							

B_m IgG D450	Vi	SE LPS	STm LPS	SE FliC	STm FliC	TT	SCh LPS
N	9	10	9	10	9	9	9
Mean	0.101	0	0	0.006	0.019	0.17	0
Median	0	0	0	0	0	0	0

B_m IgA D450	Vi	SE LPS	STm LPS	SE FliC	STm FliC	TT	SCh LPS
N	9	9	9	10	9	9	9
Mean	0.568	0.066	0.67	0.036	0	0	0
Median	0.35	0	0	0	0	0	0

Range	0,1.88	0, 0.36	0, 0	0, 0.06	0, 0.17	0, 0.73	0, 0
% Responder	22.2%	0%	0%	0%	11.1%	44.4%	0%

Range	0, 1.88	0, 0.36	0, 0.6	0, 0.36	0, 0	0, 0	0, 0
% Responder	55.6%	11.1%	11.1%	10%	0%	0%	0%

Placebo

**B_m IgG Day 1**	**Vi**	**SE LPS**	**STm LPS**	**SE FliC**	**STm FliC**	**TT**	**SCh LPS**
N	4	4	4	4	4	4	4
Mean	0	0	0	0.033	0.018	0.103	0
Median	0	0	0	0	0	0.055	0
Range	0, 0	0, 0	0, 0	0, 0.13	0, 0.07	0, 0.30	0, 0
% Responder	0%	0%	0%	25%	0%	50%	0%

**B_m IgA Day 1**	**Vi**	**SE LPS**	**STm LPS**	**SE FliC**	**STm FliC**	**TT**	**SCh LPS**
N	4	4	4	4	4	4	4
Mean	0	0	0	0	0.018	0	0
Median	0	0	0	0	0	0	0
Range	0, 0	0, 0	0, 0	0, 0	0, 0.07	0, 0	0, 0
% Responder	0%	0%	0%	0%	0%	0%	0%

**B_m IgG Day 29**	**Vi**	**SE LPS**	**STm LPS**	**SE FliC**	**STm FliC**	**TT**	**SCh LPS**
N	4	4	4	4	4	4	3
Mean	0	0	0.013	0	0.025	0.135	0.002
Median	0	0	0	0	0	0.125	0
Range	0, 0	0, 0	0, 0.05	0, 0	0, 0.1	0, 0.29	0, 0.004
% Responder	0%	0%	0%	0%	25%	25%	0%

**B_m IgA Day 29**	**Vi**	**SE LPS**	**STm LPS**	**SE FliC**	**STm FliC**	**TT**	**SCh LPS**
N	4	4	4	4	4	4	4
Mean	0	0	0.073	0	0.025	0	0
Median	0	0	0	0	0	0	0
Range	0, 0	0, 0	0, 0.29	0, 0	0, 0.1	0, 0	0, 0
% Responder	0%	0%	25%	0%	0%	0%	0%

**B_m IgG Day 57**	**Vi**	**SE LPS**	**STm LPS**	**SE FliC**	**STm FliC**	**TT**	**SCh LPS**
N	2	ns	ns	2	2	2	2
Mean	0			0	0.02	0.205	0
Median	0			0	0.02	0.205	0
Range	0, 0			0, 0	0, 0.04	0.2, 0.21	0, 0
% Responder	0%			0%	0%	50%	0%

**B_m IgA Day 57**	**Vi**	**SE LPS**	**STm LPS**	**SE FliC**	**STm FliC**	**TT**	**SCh LPS**
N	2	ns	ns	ns	ns	ns	2
Mean	0.125						0
Median	0.125						0
Range	0, 0.25						0, 0
% Responder	50%						0%

**B_m IgG D450/510**	**Vi**	**SE LPS**	**STm LPS**	**SE FliC**	**STm FliC**	**TT**	**SCh LPS**
N	2	2	2	2	2	2	2
Mean	0	0	0.04	0	0	0.09	0
Median	0	0	0.04	0	0	0.09	0
Range	0, 0	0, 0	0, 0.08	0, 0	0, 0	0, 0.18	0, 0
% Responder	0%	0%	0%	0%	0%	50%	0%

**B_m IgA D450/510**	**Vi**	**SE LPS**	**STm LPS**	**SE FliC**	**STm FliC**	**TT**	**SCh LPS**
N	2	2	2	4	4	4	2
Mean	0	0	0	0	0.05	0	0
Median	0	0	0	0	0	0	0
Range	0, 0	0, 0	0, 0	0, 0	0, 0.2	0, 0	0, 0
% Responder	0%	0%	0%	0%	25%	0%	0%

ns, no samples

<u>Antigens</u>:
Vi = *S*. Typhi Vi Polysaccharide
SE LPS = *S*. Enteritidis Lipopolysaccharide
STm LPS = *S*. Typhimurium Lipopolysaccharide
SE FliC = *S*. Enteritidis FliC flagellin subunit
STm FliC = *S*. Typhimurium FliC flagellin subunit
TT = tetanus toxoid (positive control)
SCh LPS = *S*. Choleraesuis Lipopolysaccharide (negative control)

Figure 7. Gating Protocol of Sorted B cell subpopulations

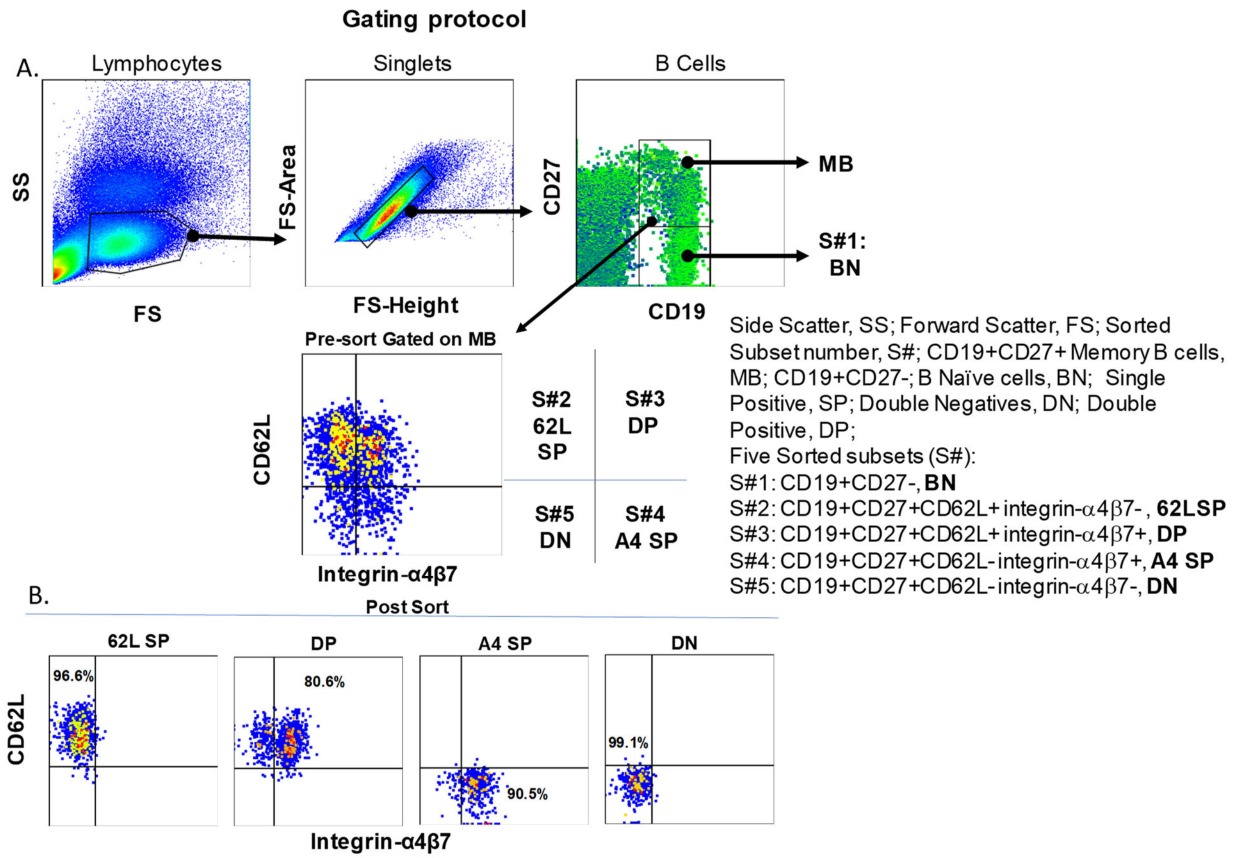

Representative gating protocol for B cell sorting. Freshly isolated PBMC purified from subjects 7-days after vaccination with TSCV (Day 8) were stained with monoclonal antibodies against CD19, CD27, CD62L, and integrin α4β7 markers and the stained cells were simultaneously sorted into five different B cell subsets, using a MoFlow Astrios cell sorter (Beckman-Coulter) as described in the methods section. Sequential gating protocols are shown in **panel A**. An aliquot of B Memory (BM) cell sorted populations was analyzed post-sorting to assess purity of the subsets **(panel B).**

Figure 8. Percentage of antigen-specific BM responses

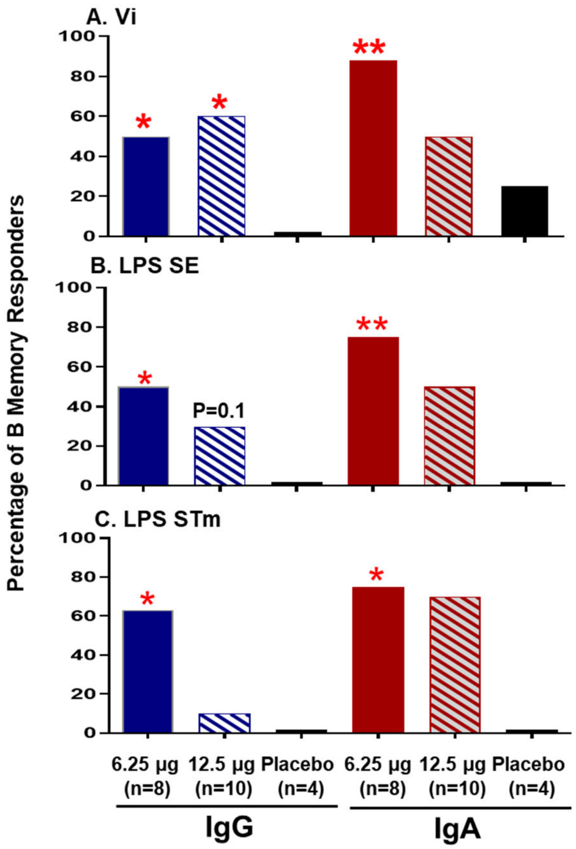

Percentages of responders showing increased antigen-specific B memory responses to polysaccharide antigens in TSCV following vaccination. Shown are the percentages of responders for the induction of IgG and IgA BM responses specific for Vi from *S*. Typhi (**panel A**), LPS from *S*. Enteritidis - LPS SE (panel B) and LPS from *S*. Typhimurium - STm (**panel C**), following immunization with 6.25 µg or 12.5 µg TSCV or Placebo. A cutoff value for antigen specific responses was calculated as the Mean+3 SE of the corresponding antigen specific IgG and IgA BM responses in pre-vaccination (D1) samples from all participants (n=22). Volunteers showing post-vaccination increases above the corresponding antigen specific pre-vaccination cut-off values at any of the post-vaccination time points (Day 29, Day 57 or days 450/510) were considered responders. P values were determined by comparing vaccinated volunteers with the corresponding antigens in the placebo group (n=4) using Chi square tests. *p<0.05, ** p<0.01.
